# Pre-clinical Evidence: Berberine as a Promising Cardioprotective Candidate for Myocardial Ischemia/Reperfusion Injury, a Systematic Review, and Meta-Analysis

**DOI:** 10.3389/fcvm.2021.646306

**Published:** 2021-05-26

**Authors:** Cong Chen, Qian Lin, Xue-Ying Zhu, Junyan Xia, Tianshi Mao, Tiange Chi, Jie Wan, Jin-Jin Lu, Yan Li, Jie Cui, Jing Liu, Xiao-Yun Cui, Jingqian Zhang, Kun Zhou, Dong Li

**Affiliations:** ^1^Department of Cardiology, Dongzhimen Hospital Affiliated to Beijing University of Chinese Medicine, Beijing, China; ^2^Department of Cardiology, Dongfang Hospital Affiliated to Beijing University of Chinese Medicine, Beijing, China; ^3^First Clinical Medical School, Beijing University of Chinese Medicine, Beijing, China

**Keywords:** pre-clinical evidence, molecular mechanisms, systematic review, berberine, myocardial ischemia/reperfusion injury

## Abstract

**Objective:** Myocardial ischemia/reperfusion (I/R) injury is one of the causes of most cardiomyocyte injuries and deaths. Berberine (BBR) has been suggested a potential to exert protective effects against myocardial I/R injury. This systematic review aims to determine the intrinsic mechanisms of BBR's protective effects in myocardial I/R injury.

**Methods:** Seven databases were searched for studies performed from inception to July 2020. Methodological quality was assessed by SYRCLE's-RoB tool.

**Results:** Ten studies including a total of 270 animals were included in this study. The methodology quality scores of the included studies ranged from 5 to 7 points. The meta-analysis we conducted demonstrated that BBR significantly reduced myocardial infarct size and the incidence of ventricular arrhythmia, compared to control groups (*P* < 0.00001). Cardiac function of animals in the BBR treatment group was also markedly increased (*P* < 0.00001). The index of myocardial apoptosis and the levels of biomarkers of myocardial infarction (LDH and CK) were also decreased in the BBR treatment groups compared to the control groups (*P* < 0.00001).

**Conclusions:** The pre-clinical evidence, according to our study, showed that BBR is a promising therapeutic agent for myocardial I/R injury. However, this conclusion should be further investigated in clinical studies.

## Introduction

Despite recent progress in the diagnosis and treatment of cardiovascular diseases, acute myocardial infarction (AMI) remains a leading cause of death and disabilities worldwide (1, 2). Percutaneous coronary intervention (PCI) is one of the most effective reperfusion strategies for AMI (3). However, the process of myocardial reperfusion can itself induce further cardiomyocyte death, a phenomenon known as myocardial ischemia/reperfusion (I/R) injury (4). The exact mechanisms of myocardial I/R injury are not fully understood (5). What is known is the pathological process of I/R injury, which begins with inflammation, oxidative stress and intracellular Ca2+ overload, taking place in a time-dependent fashion. Eventually, irreversible cell death is caused by apoptosis and necrosis (6). Myocardial I/R injury presents in various forms, including reperfusion-induced arrhythmias, myocardial stunning, and microvascular obstruction. In particular, ischemia-induced lethal ventricular arrhythmias (VAs) can induce sudden death (7). Therefore, novel strategies to improve myocardial salvage and cardiac function call for further investigation.

**Figure 1:**
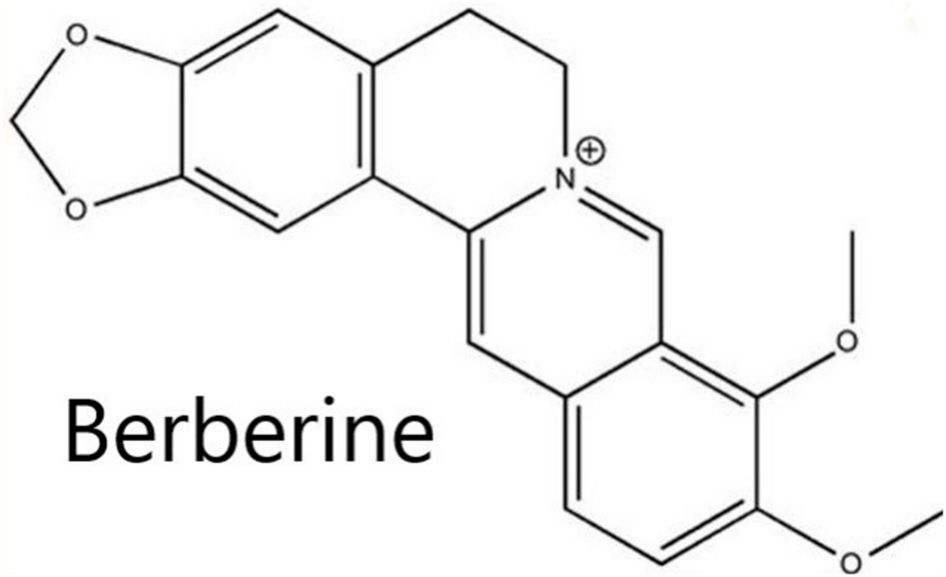
Molecular structure of BBR.

BBR (Figure 1), a natural isoquinoline alkaloid, extracted from the Chinese medicinal *Coptis chinensis Franch* (also named “Huang Lian” in Chinese) (8). Huang Lian was first recorded as a medicinal in the book “Shen Nong Ben Cao Jing,” dated A.D. 200, which was used to treat inflammatory disorders (9). BBR presents in a form of a yellow odorless powder, with characteristic alkaloid bitterness and a molar weight of 336.36 g/mol (10). The salt form of BBR is relatively water-soluble (11). BBR can be easily obtained, either from the extraction from medicinal plants or artificial synthesis (12). Because of its antiatherosclerotic, lipid-lowering, antiobesity, and antihepatic steatosis effects, BBR might presents as a promising cardioprotective candidate for myocardial ischemia/reperfusion injury (9). However, no relevant studies that systematically evaluate the mechanisms of BBR in an experimental myocardial I/R injury model are available.

## Methods

### Search Strategy

Seven databases, including Medline, EMBASE, Cochrane Library, China National Knowledge Infrastructure (CNKI), Wangfang database, VIP database (VIP), and China Biology Medicine disc (CBM), were systematically searched from inception to July 2020. The keywords “myocardial infarction OR myocardial ischemia OR myocardial I/R OR myocardial I/R injury OR ischemia/reperfusion injury OR myocardial ischemia/reperfusion injury” AND “berberine (MeSH Terms) OR berberine (Title/Abstract)” were retrieved. Moreover, commentary articles, including meeting abstracts and reference review articles, were scrutinized. All included studies were limited to animals, and there were no language restrictions.

### Inclusion and Exclusion Criteria

To prevent bias, the inclusion criteria were prespecified as follows: (1) The I/R experimental model is established by ligating the left anterior descending (LAD) coronary artery or injecting vasoconstrictor to intravenously; (2) BBR is the only means of intervention, and control animals received placebo fluids or no treatment at all; (3) non-human studies; and (4) the data on the levels of MI size (Evans blue/TTC staining or only TTC staining for *ex vivo* studies), cardiac enzymes, biomarkers of myocardial injury, and left ventricular ejection fraction (LVEF) were collected for outcome evaluation. The following were prespecified exclusion criteria: (1) no model animals; (2) BBR is not the only intervention; (3) no control group; (4) repeated literature; (5) combined with cardiovascular comorbidity; and (6) *in vitro* studies.

### Data Extraction

Two reviewers collected data independently, and a third reviewer resolved any divergence. The standardized predefined forms for data extraction were as follows: (1) first author name and publication year; (2) animal data, including species, sex, and body weight/age; (3) model preparation and anesthesia methods; (4) group information, including the dosage of drug, method of administration and duration of intervention; and (5) mean and standard deviation of the results. When there were many different time point outcomes, only the result of the peak time point was recorded. When the experimental animals received different doses of the drug, only the highest dose was recorded. Some outcome measures were published in graphical format. We used digital ruler software to measure numerical values from the graphs when we were unable to contact the authors for further information (13).

### Risk of Bias

The quality of the included studies was assessed using SYRCLE's RoB tool 14. Two reviewers who performed data extraction separately evaluated the risk of bias in the included studies. Each of the following was scored as one point: (a) sequence generation; (b) baseline characteristics; (c) allocation concealment; (d) random housing; (e) blinding of the investigator; (f) random outcome assessment; (g) blinding of the outcome assessor; (h) incomplete outcome data; (i) selective outcome reporting; and (j) other sources of bias.

### Statistical Analyses

Myocardial infarct size, cardiac function, the incidence of ventricular arrhythmia, the index of myocardial apoptosis, and biomarkers of myocardial infarction were analyzed using Cochrane Program Review Manager Version 5.3 (Cochrane Collaboration, Oxford, UK). The *I*^2^-statistic test was used to evaluate heterogeneity. Outcomes used a random-effects model to decrease heterogeneity; otherwise, a fixed-effects model was applied. Outcomes are presented as risk ratios (RRs) and weighted mean differences (WMDs) with 95% confidence intervals (CIs). Publication bias was assessed by funnel plots, and meta-regression analysis was used to evaluate factors that affected the heterogeneity.

## Results

### Study Selection

**Figure 2:**
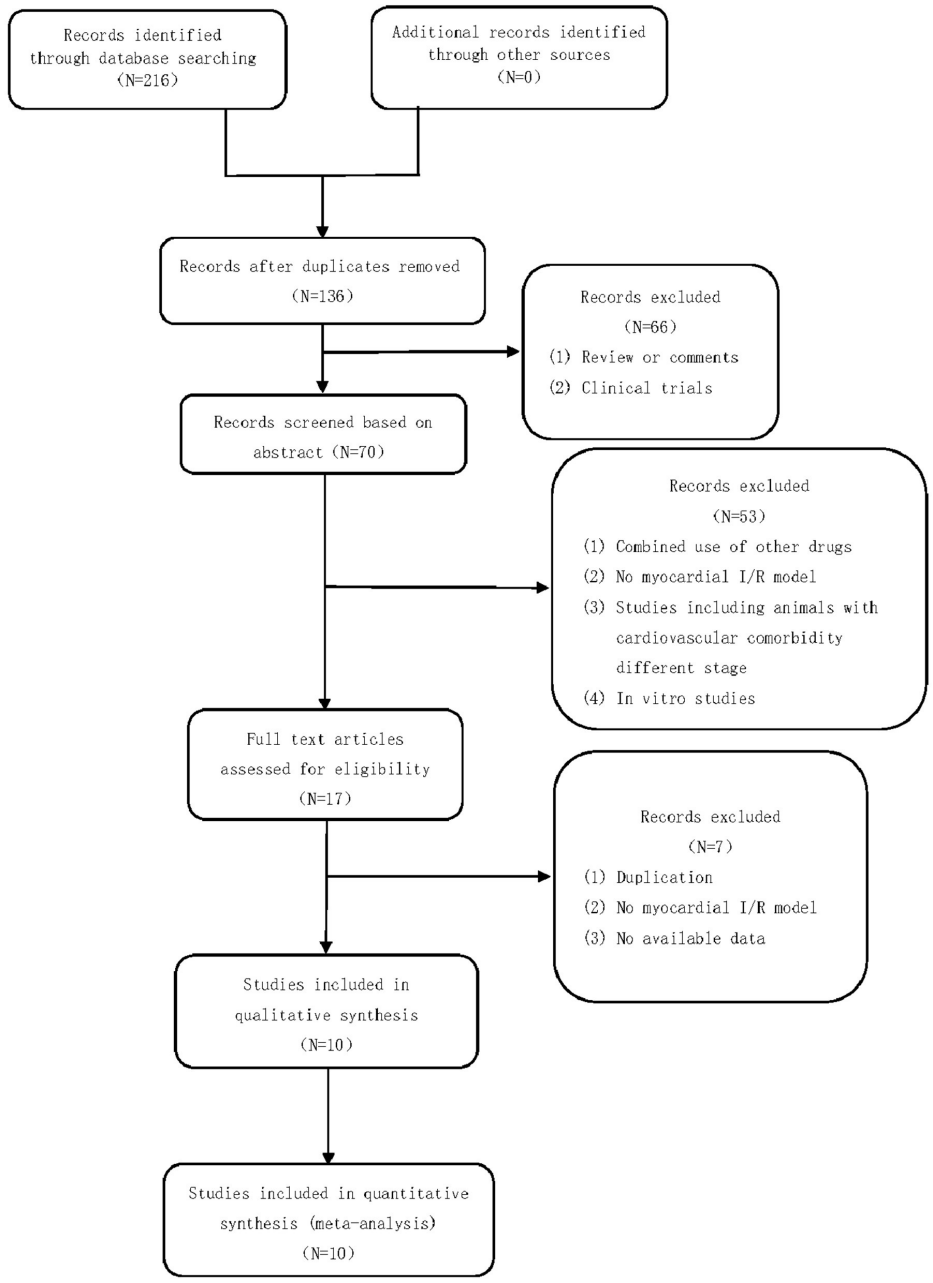
Summary of the process for identifying candidate studies.

A total of 216 studies were identified by searching the databases. After removing duplicates and irrelevant studies, 136 studies remained. After screening the titles and abstracts, 70 studies remained. A total of 66 articles were excluded for the following reasons: clinical trials, comments, or reviews. After reading the full text of the articles, 17 studies were included. Fifty-three studies were excluded for the following reasons: (1) combined use of other drugs, (2) no myocardial I/R model, (3) studies including animals with cardiovascular comorbidity of different stages, and (4) *in vitro* studies. Of the 17 studies that were included in the qualitative synthesis, we excluded 7 studies for the following reasons: (1) duplication, (2) no myocardial I/R model, and (3) no available data. Finally, 10 studies (14–22) were selected. The study selection process is summarized in a flow chart (Figure 2).

### Characteristics of Included Studies

A total of 10 studies (14–23) with 270 animals was included between 2009 and 2020. All studies were conducted in China, including 7 studies (14, 15, 17, 19, 20, 22, 23) in English and 3 studies (16, 18, 21) in Chinese [among these studies, two were online master's theses (16, 21) in Chinese]. Male or female Sprague-Dawley rats, male Wistar rats, and male mouse animal models were used in 6 studies (15, 17–21), 3 studies (16, 22, 23), and 1 study (14), respectively. The weight of adult rats varied from 180 grams to 300 g, and that of the mice used varied from 20 to 25 g. All myocardial infarction models were produced by ligation of the LAD. Five studies (15, 17, 19–21) used sodium pentobarbital, 2 studies (18, 22) used chloral hydrate, 2 studies (16, 23) used urethane, and 1 study (14) used isoflurane. The dose of BBR in the rat model varied between 50 and 300 mg·kg^−1^·d^−1^, but the mice model was pre-conditioned with 10 mg·kg^−1^·d^−1^ BBR by intraperitoneal injection in one study (14). Nine studies (14, 16–23) utilized the myocardial infarct size assay as an outcome measure. Five studies (14, 19–21, 23) used echocardiography to evaluate cardiac function (LVEF, LVFS, LVESD, LVEDD, and LVDP as outcome measures). Six studies (17–22) analyzed myocardial apoptosis by measuring the myocardial cell apoptosis rate. Six studies (17–22) detected biomarkers of myocardial injury by determining levels of serum creatine kinase (CK), creatine kinase MB (CK-MB), lactate dehydrogenase (LDH), aspartate aminotransferase (ASK), and cardiac troponin I activity (cTnI). Two studies (15, 20) measured cardiomyocyte inflammatory mediators by detecting levels of tumor necrosis factor (TNF)-α, interleukin (IL)-6, and IL-1β. Three studies (16, 20, 21) measured myocardial superoxide generation, gp91phox protein expression, MDA concentration, and SOD activity. Three studies (15, 16, 23) investigated ventricular arrhythmia following I/R insult. The overall characteristics of the included publications are shown in Table 1.

**Table 1:** Characteristics of included studies.

**References**	**Country**	**Species (sex, *n* = experimental/control group)**	**Weight/year**	**Model (method)**	**Anesthetic**	**Treatment group (method to astragal sides)**	**Control group**	**Duration, week**	**Outcomes**	**Intergroup differences**
Yu et al. (20)	China	SD rats (male 10/10)	250–300 g	Block LAD for 30 min then reflow for 4, 6, 24 h	Pentobarbital sodium 3%	BBR (dissolved in 0.5% CMC-Na solution) oral 200 mg/kg/d	0.5% CMC-Na solution oral 2 mL	2	(1) LVEF (2) LVFS (3) Myocardial infarct size (4) Myocardial cell apoptosis rate (5) CK (6) LDH (7) gp91^phox^ protein (8) MDA (9) SOD (10) IL-6 (11) TNF-α (12) MPO	(1) *P* < 0.01 (2) *P* < 0.01 (3) *P* < 0.01 (4) *P* < 0.01 (5) *P* < 0.01 (6) *P* < 0.01 (7) *P* < 0.01 (8) *P* < 0.01 (9) *P* < 0.01 (10) *P* < 0.01 (11) *P* < 0.01 (12) *P* < 0.01
Wang et al. (17)	China	SD rats (male 20/20)	250–300 g	Block LAD for 30 min then reflow for 3 h	Pentobarbital sodium 40 mg/kg	BBR oral 200 mg/kg/d	Saline	4	(1) Myocardial infarct size (2) CK-MB (3) cTnI (4) LDH (5) Myocardial cell apoptosis rate (6) MMP (7) Mitochondrial complex I	(1) *P* < 0.05 (2) *P* < 0.05 (3) *P* < 0.05 (4) *P* < 0.05 (5) *P* < 0.05 (6) *P* < 0.05 (7) *P* < 0.05
Zhu et al. (22)	China	Wistar rats (male 5/5)	180–200 g	Block LAD for 30 min then reflow for 2 h	Chloral hydrate 10%	BBR oral 300 mg/kg/d	NM	3 days	(1) Myocardial infarct size (2) CK-MB (3) AST (4) LDH (5) Myocardial cell apoptosis rate	(1) *P* < 0.05 (2) *P* < 0.05 (3) *P* < 0.05 (4) *P* < 0.05 (5) *P* < 0.05
Qin-Wei and Yong-Guang (15)	China	SD rats (male 10/10)	200–250 g	Block LAD for 30 min then reflow for 4 h	Pentobarbital sodium 1%	BBR oral 100 mg/kg/d	Saline	2	(1) Incidence of ventricular arrhythmia (2) TNF-α (3) IL-6 (4) IL-1β	(1) *P* < 0.05 (2) *P* < 0.01 (3) *P* < 0.05 (4) *P* < 0.01
Yu et al. (19)	China	SD rats (male 20/20)	220–250 g	Block LAD for 30 min then reflow for 4, 6, 72 h	Pentobarbital sodium 3%	BBR (dissolved in 0.5% CMC-Na solution) oral 200 mg/kg/d	0.5% CMC-Na solution oral 2 mL	2	(1) LVEF, (2) LVFS (3) Myocardial infarct size (4) Myocardial cell apoptosis rate (5) CK (6) LDH	(1) *P* < 0.01 (2) *P* < 0.01 (3) *P* < 0.01 (4) *P* < 0.01 (5) *P* < 0.01 (6) *P* < 0.01
Xiong and Wei (18)	China	SD rats (male/female 8/8)	180–200 g	Block LAD for 30 min then reflow for 1.5 h	Chloral hydrate 10%	BBR (dissolved in 0.5% CMC-Na solution) oral 300 mg/kg/d	0.5% CMC-Na solution oral	1	(1) Myocardial infarct size (2) LDH (3) CPK (4) Myocardial cell apoptosis rate	(1) *P* < 0.01 (2) *P* < 0.01 (3) *P* < 0.01 (4) *P* < 0.01
Zhao et al. (21)	China	SD rats (male 8/8)	200–250 g	Block LAD for 30 min then reflow for 2 h	Pentobarbital sodium 3%	BBR (dissolved in 0.5% CMC-Na solution) oral 200 mg/kg/d	0.5% CMC-Na solution oral	3	(1) LVEF (2) LVFS (3) Myocardial infarct size (4) Myocardial cell apoptosis rate (5) CK (6) LDH (7) gp91phox protein	(1) *P* < 0.05 (2) *P* < 0.05 (3) *P* < 0.05 (4) *P* < 0.05 (5) *P* < 0.05 (6) *P* < 0.05 (7) *P* < 0.05
Song (16)	China	Wistar rats (male 15/15)	250–300 g	Block LAD for 30 min then reflow for 2 h	Urethane 20%	BBR oral 50 mg/kg/d	Saline	10 days	(1) Myocardial infarct size (2) SOD (3) MDA (4) Incidence of ventricular arrhythmia	(1) *P* < 0.01 (2) *P* < 0.05 (3) *P* < 0.01 (4) *P* < 0.05
Huang et al. (14)	China	C57BL/6 mice (male 28/20)	20–25 g	Block LAD for 30 min then reflow for 3 h	Isoflurane 4%	BBR Intravenous injection 10 mg/kg	Saline	15 min	(1) Myocardial infarct size (2) LVESD (3) LVEDD (4) FS (5) EF	(1) *P* < 0.05 (2) *P* < 0.05 (3) *P* < 0.05 (4) *P* < 0.05 (5) *P* < 0.05
Chang et al. (23)	China	Wistar rats (male 15/15)	250–280 g	Block LAD for 30 min then reflow for 2 h	Urethane 20%	BBR oral 100 mg/kg/d	Water	14 days	(1) LVDP (2) LVEDP (3) Incidence of ventricular arrhythmia (4) Myocardial infarct size	(1) *P* < 0.05 (2) *P* < 0.05 (3) *P* < 0.05 (4) *P* < 0.05

*BBR, Berberine; SD rats, Sprague-Dawley; NM, no mention; LVEF, Left ventricular ejection fraction; LVFS, left ventricular fractional shortening; LVESD, left ventricular end-systolic diameter; LVEDD, left ventricular end-diastolic diameter; LVEDP, left ventricular end-diastolic pressure; LVDP, left ventricular developed pressure; FS, Fractional shortening; EF, ejection fraction; CK, creatine kinase; LDH, lactate dehydrogenase; MDA, malondialdehyde; SOD, superoxide dismutase; CK-MB, creatine kinase-MB; cTnI, cardiac troponin I activity; MMP, mitochondrial membrane potential; CPK, Creatine Phosphokinase; TNF-α, tumor necrosis factor (TNF)-α; IL-6, interleukin (IL)-6; MPO, myeloperoxidase*.

### Methodological Quality of the Included Studies

The quality scores of the studies ranged from 5 to 7 points. Two studies (15, 17) received 7 points, 6 studies (14, 18–20, 22, 23) received 6 points, and 2 studies (16, 21) received 5 points. All included studies were at low risk of bias regarding baseline characteristics, allocation concealment, random outcome assessment, and incomplete outcome data. No studies described sequence generation, blinding investigators, or blinding outcome assessors. Four studies (15, 17, 18, 22) described random housing. Seven studies (14, 15, 17, 19, 20, 22, 23) exhibited selective outcome reporting, and 9 studies (14–21, 23) declared no other sources of bias. The details of the methodological quality are shown in Table 2.

**Table 2:** Quality assessment of included randomized controlled trials.

**References**	**A**	**B**	**C**	**D**	**E**	**F**	**G**	**H**	**I**	**J**	**Scores**
Yu et al. (20)	0	1	1	0	0	1	0	1	1	1	6
Wang et al. (17)	0	1	1	1	0	1	0	1	1	1	7
Zhu et al. (22)	0	1	1	1	0	1	0	1	1	0	6
Qin-Wei and Yong-Guang (15)	0	1	1	1	0	1	0	1	1	1	7
Yu et al. (19)	0	1	1	0	0	1	0	1	1	1	6
Xiong and We (18)	0	1	1	1	0	1	0	1	0	1	6
Zhao et al. (21)	0	1	1	0	0	1	0	1	0	1	5
Song (16)	0	1	1	0	0	1	0	1	0	1	5
Huang et al. (14)	0	1	1	0	0	1	0	1	1	1	6
Chang et al. (23)	0	1	1	0	0	1	0	1	1	1	6

*(A) Sequence generation; (B) Baseline characteristics; (C) Allocation concealment; (D) Random housing; (E) Blinding investigators; (F) Random outcome assessment; (G) Blinding outcome assessor; (H) Incomplete outcome data; (I) Selective outcome reporting; and (J) Other sources of bias*.

*1, low risk of bias: the information of the domain was adequate in the text; 0, high risk or unclear of bias: the information of the domain was inadequate in the text*.

## Outcome Measures

### Myocardial Infarct Size

**Figure 3:**
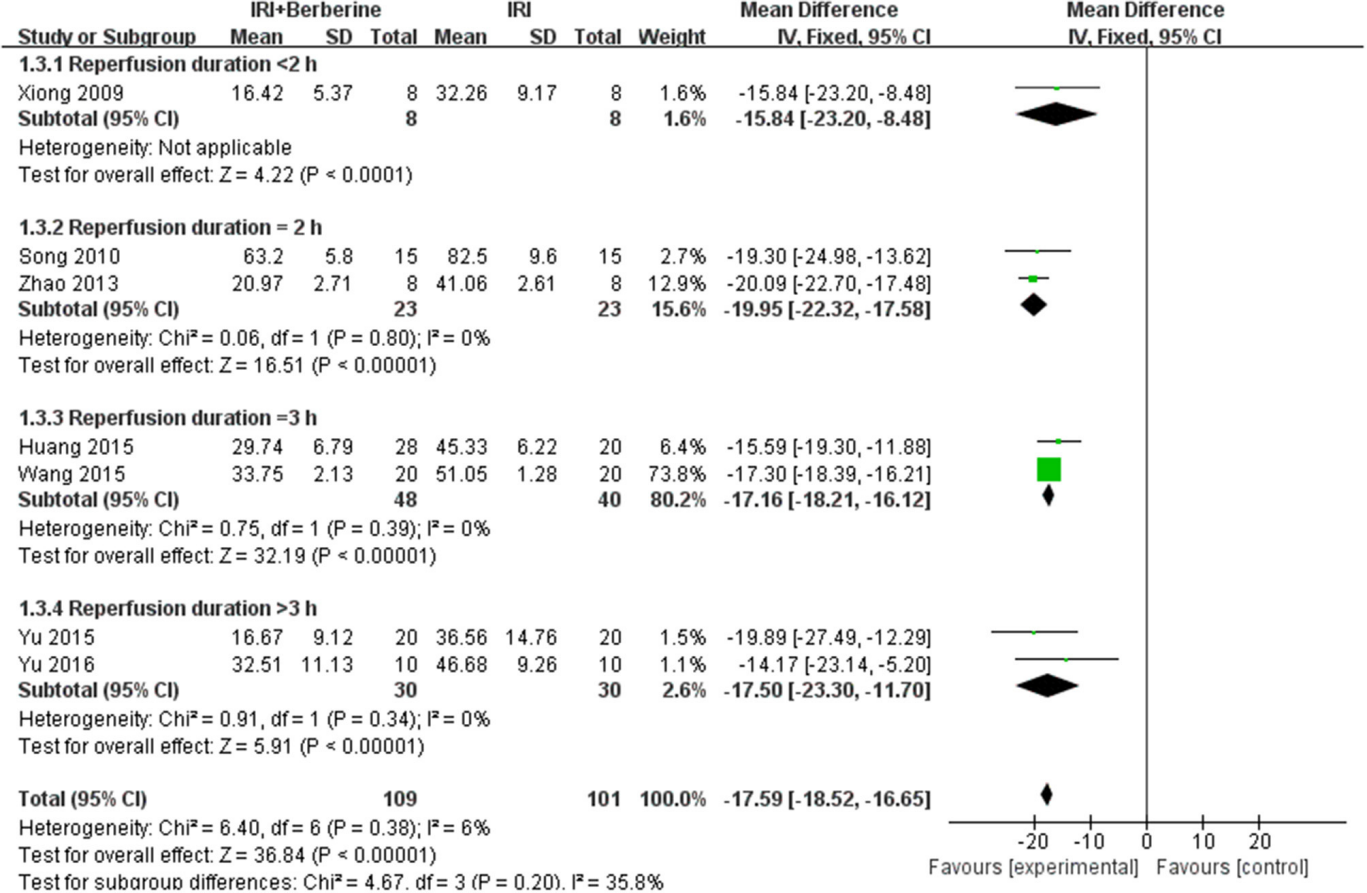
Forest plot: effects of BBR on decreasing the myocardial infarction size compared with the control group.

**Figure 4:**
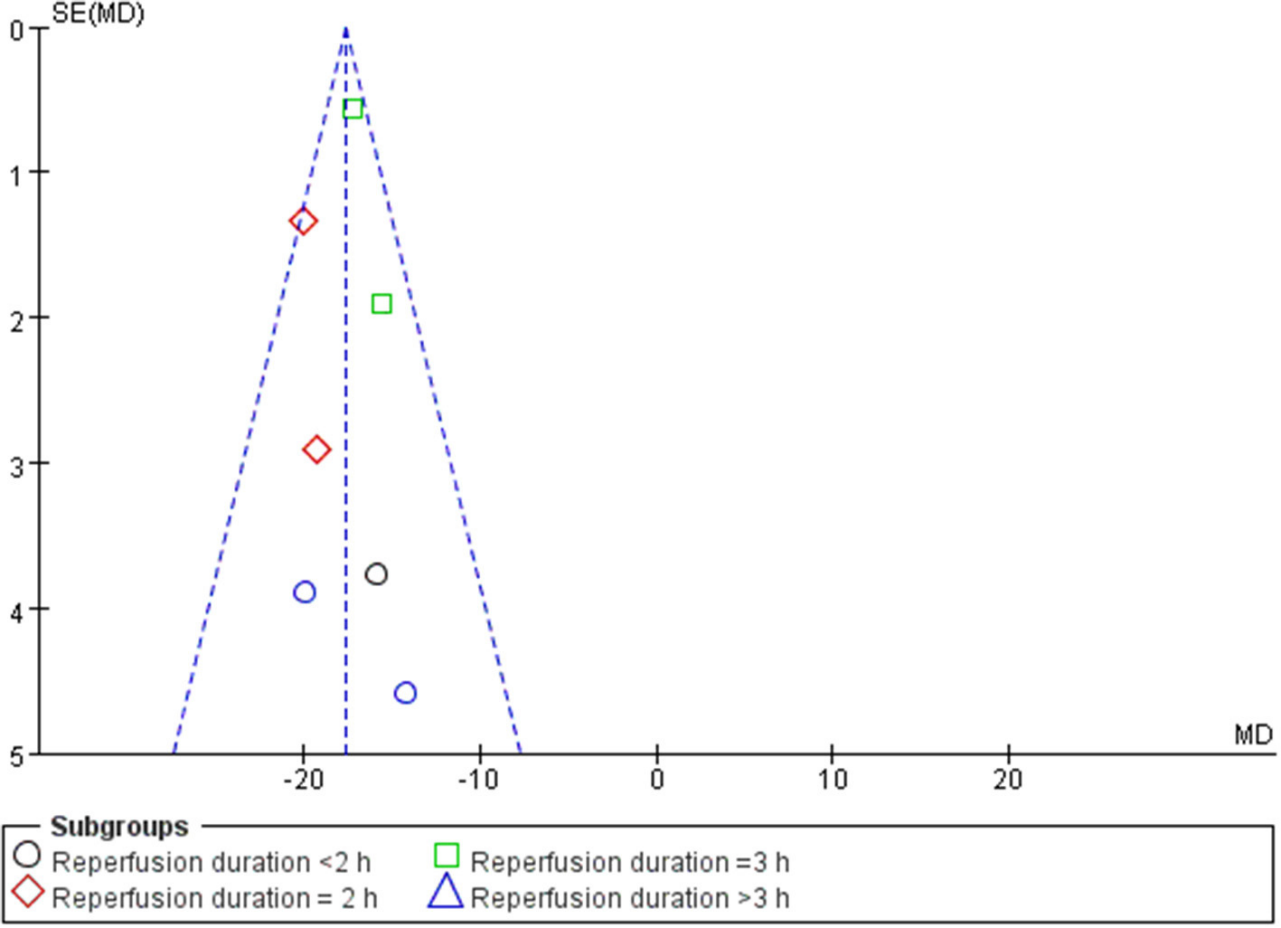
Funnel plot showing BBR vs. control group regarding myocardial infarction size.

A meta-analysis of 9 studies (14, 16–23) demonstrated that BBR significantly decreased myocardial infarct (MI) size compared to the control group [*n* = 250, *MD* = −14.03, 95% CI (−14.85, −13.21), *P* < 0.00001, *I*^2^ = 97%]. Because of obvious heterogeneity in the included studies, we used sensitivity analyses. Two studies (22, 23) were the origin of obvious heterogeneity, which was determined by systematically excluding each study. After removing these studies, there was no significant heterogeneity among the remaining research. Thus, a fixed effects model was used for statistical analysis. A meta-analysis of 7 studies (14, 16–21) showed a significant effect of BBR on decreasing MI size compared to the control groups [*n* = 210, *MD* = −17.59, 95% CI (−18.52, −16.65), *P* < 0.00001, *I*^2^ = 6%]. Notably, subgroup analysis according to reperfusion duration suggested that the pooled estimates for decreasing MI size did not depend on it (Figure 3). The funnel plot did not show any asymmetry, demonstrating the absence of publication bias (Figure 4). Meta-regression did not reveal a significant impact of covariates (i.e., species, sample size, and reperfusion duration) on the decrease in MI size of BBR (Table 3).

**Table 3:** Meta-regression analysis of potential sources of heterogeneity.

**Heterogeneity factor**	**Coefficient**	**SE**	**Z**	***P-*value**	**95% CI (lower limit, upper limit)**
Reperfusion duration	1.477604	1.817508	0.81	0.476	−4.306517, 7.261725
Species	2.888427	2.793626	1.03	0.377	−6.002138, 11.77899
Sample size	−0.175114	0.1942815	−0.9	0.434	−0.7934044, 0.4431763
Cutoff value	−6.684941	5.174406	−1.29	0.287	−23.15221, 9.782329

*CI, confidence interval*.

### Cardiac Function

**Figure 5:**
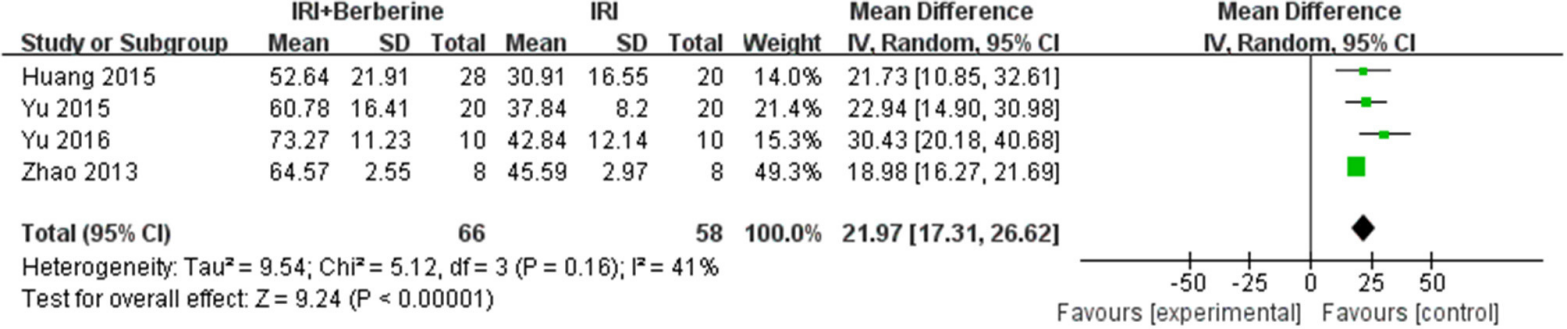
Forest plot showing the effects of BBR on increasing LVEF compared to the control group.

**Figure 6:**
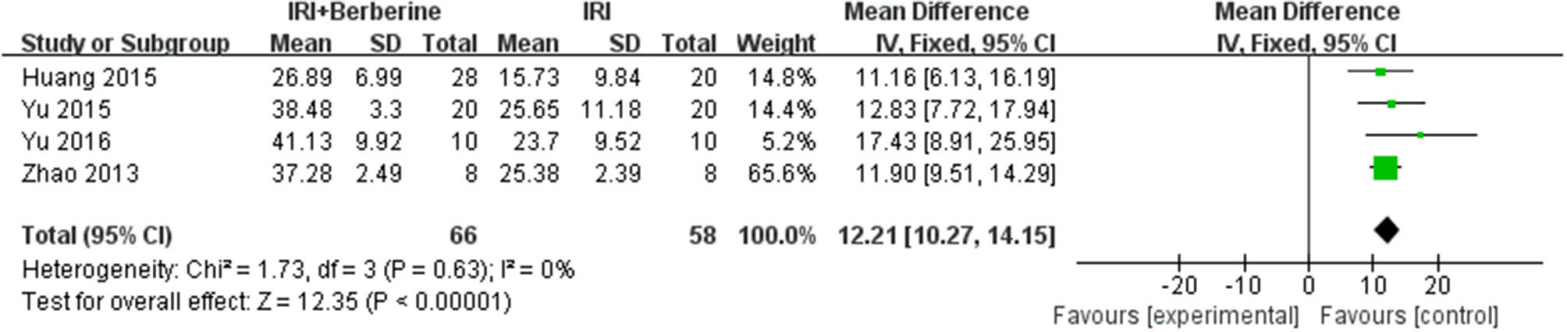
Forest plot showing the effects of BBR on increasing LVFS compared to the control group.

Left ventricular ejection fraction (LVEF) and left ventricular fractional shortening (LVFS) were examined to demonstrate improvements in cardiac function induced by BBR. A total of 4 trials (14, 19–21) reported the protective effects of BBR on LVEF and LVFS compared to the control groups. The 4 independent studies (14, 19–21) of LVEF showed significant heterogeneity. Thus, a random effects model was used for statistical analysis. The meta-analysis demonstrated that BBR led to a greater increase in LVEF than in the control groups [*n* = 124, *MD* = 21.97, 95% CI (17.31, 26.62), *P* < 0.00001, *I*^2^ = 41%] (Figure 5). However, the meta-analysis of 4 LVFS studies (14, 19–21) did not show significant heterogeneity. Thus, in the pooled analysis using a fixed effects model, preconditioning with BBR markedly increased LVFS compared to the control groups [*n* = 124, *MD* = 12.21, 95% CI (10.27, 14.15), *P* < 0.00001, *I*^2^ = 0%] (Figure 6).

### The Incidence of Ventricular Arrhythmia

**Figure 7:**
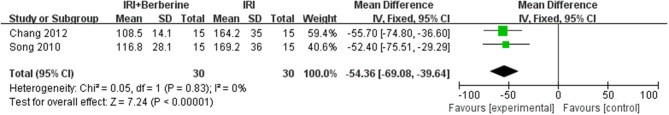
Forest plot showing the effects of BBR on decreasing pre-mature ventricular contraction compared to the control group.

**Figure 8:**
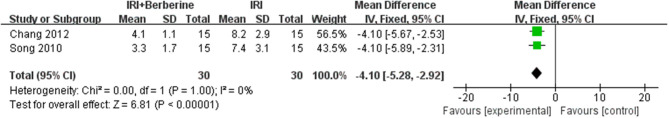
Forest plot showing the effects of BBR on decreasing the cumulative duration of ventricular tachycardia compared to the control group.

**Figure 9:**
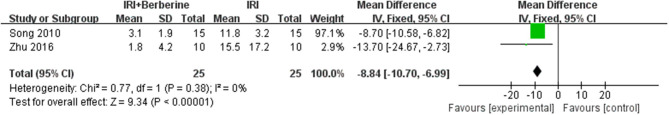
Forest plot showing effects of BBR on decreasing the cumulative duration of ventricular fibrillation in the control group.

Three studies (15, 16, 23) investigated the effects of BBR on ventricular arrhythmia following I/R injury. After sensitivity analysis, we removed the study with data that showed obvious heterogeneity. A meta-analysis of 2 studies (16, 23) using a fixed effects model showed a significant effect of BBR on decreasing the counts of premature ventricular contraction (PVC) compared to the control groups [*n* = 60, *MD* = −54.36, 95% CI (−69.08, −39.64), *P* < 0.00001, *I*^2^ = 0%] (Figure 7). A meta-analysis of 2 studies (16, 23) using a fixed effects model showed that BBR markedly diminished the cumulative duration of ventricular tachycardia (VT) compared to the control groups [*n* = 60, *MD* = −4.1, 95% CI (−5.28, −2.92), *P* < 0.00001, *I*^2^ = 0%] (Figure 8). A meta-analysis of 2 studies (15, 16) using a fixed effects model showed that BBR markedly diminished the cumulative duration of ventricular fibrillation (VF) compared to the control groups [*n* = 50, MD = −8.84, 95% CI (−10.7, −6.99), *P* < 0.00001, *I*^2^ = 0%] (Figure 9).

### Myocardial Apoptosis

**Figure 10:**
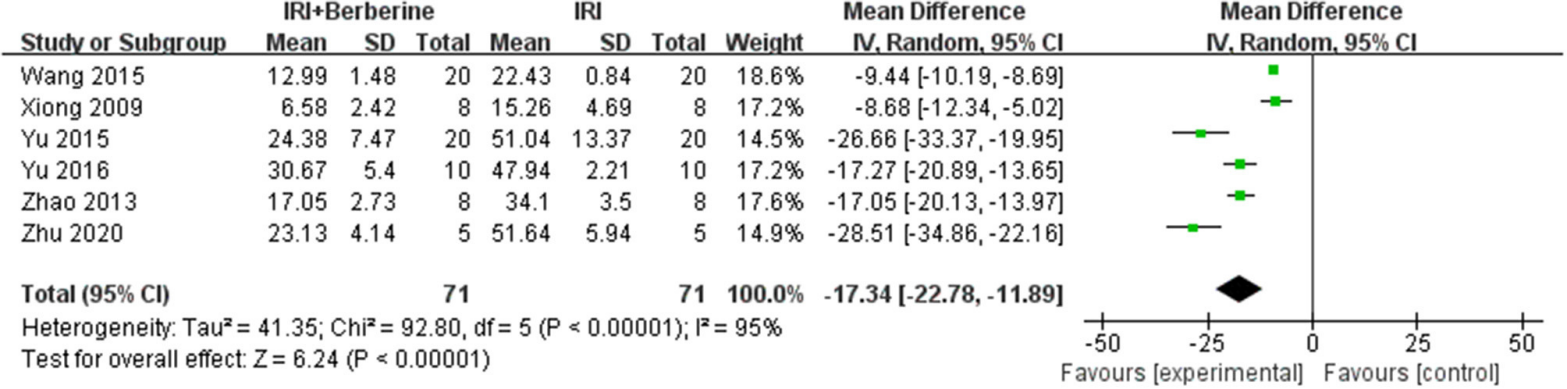
Forest plot showing effects of BBR on decreasing the index of myocardial apoptosis compared with the control group.

Six studies utilized myocardial apoptosis level as the outcome measure. All included studies used a terminal deoxynucleotidyl transferase-mediated dUTP nick end labeling (TUNEL) assay to analyze the levels of myocardial apoptosis. The level of myocardial apoptosis were evaluated by the counts of apoptotic cardiomyocytes or the total number of cardiomyocytes counted × 100%. A meta-analysis of these studies showed significant effects of BBR in decreasing the level of myocardial apoptosis compared to the control groups [*n* = 142, *MD* = −17.34, 95% CI (−22.78, −11.89), *P* < 0.00001, *I*^2^ = 95%] (Figure 10). There was significant heterogeneity among the studies. Therefore, a random effects model was used for the meta-analysis.

### Serum LDH and Serum CK Levels

**Figure 11:**
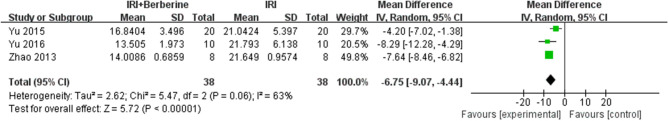
Forest plot showing the effects of BBR on decreasing LDH compared to the control group.

**Figure 12:**
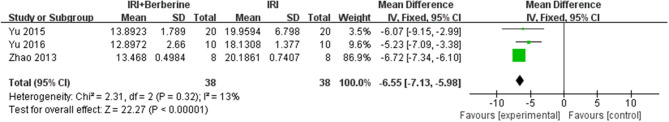
Forest plot showing the effects of BBR on decreasing CK compared to the control group.

Six studies reported LDH as an outcome measure. However, the data of LDH was not available in one study and it was since removed. Meta-analysis of 5 studies showed a significant effect of BBR on decreasing LDH compared to the control groups [*n* = 102, *MD* = −7.57, 95% CI (−8.33, −6.8), *P* < 0.00001, *I*^2^ = 97%]. After sensitivity analysis, we removed 2 studies that caused obvious heterogeneity. BBR in the remaining 3 studies showed significant effects on reducing LDH compared to the control groups [*n* = 76, *MD* = −6.75, 95% CI (−9.07, −4.44), *P* < 0.00001, *I*^2^ = 63%] (Figure 11). A meta-analysis of 3 studies showed a significant effect of BBR on decreasing CK compared to the control groups [*n* = 76, *MD* = −6.55, 95% CI (−7.13, −5.98), *P* < 0.00001, *I*^2^ = 13%] (Figure 12). Due to low heterogeneity among the trials, the fixed effects model was used for the meta-analysis.

## Discussion

### Summary of Evidence

This is the first preclinical meta-analysis and systematic review to investigate the cardioprotective effects of BBR on myocardial I/R injury. Ten studies with 270 animals were included. The methodological quality of the included studies was generally moderate. The findings of our study indicated that, compared to vehicle control, BBR reduces myocardial infarct size and the incidence of ventricular arrhythmia, improves cardiac function, ameliorates myocardial apoptosis, and decreasing biomarker levels of myocardial infarction.

### Molecular Mechanisms

**Figure 13:**
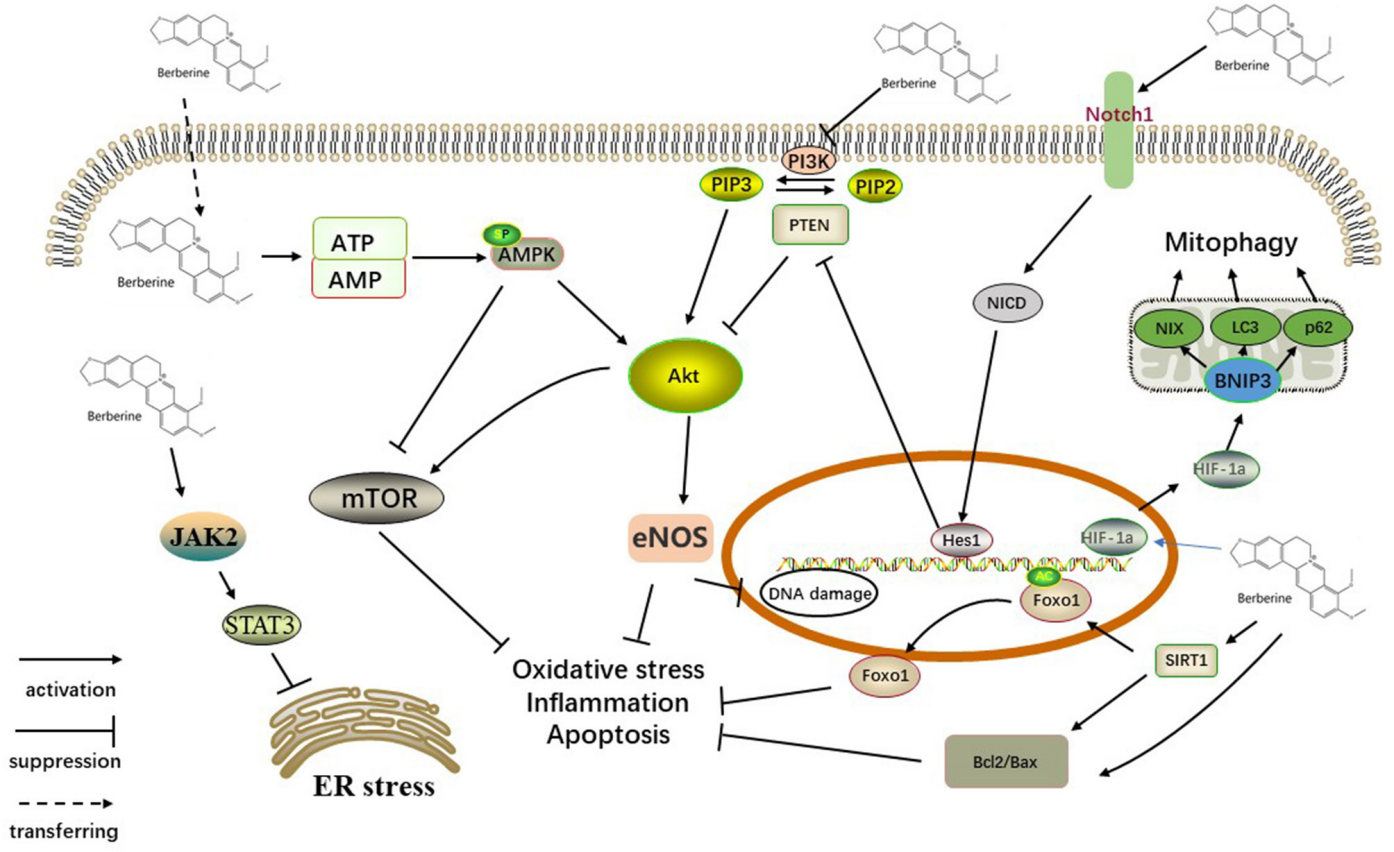
Schematic representation of BBR in cardiac myocytes. BBR upregulated expression of NICD and Hes1 by activating Notch1, which is distributed on the cell membrane. PTEN/Akt, the downstream signaling pathway, also participates in this process. Activation of the SIRT1/FOXO signaling pathway might also play a key role in the antiapoptotic action of BBR. BBR activates AMPK and other pathways that inhibited the Akt pathway, further acting on the mTOR factor. In addition, activating JAK2/STAT3 signaling participates in the action of BBR on ER stress. BBR also promotes mitophagy via the HIF-1α/BNIP3 pathway. SIRT1, silent information regulator 1; FOXO, forkhead box O; ER, endoplasmic reticulum stress, JAK, Janus kinase; STAT, signal transducer and activator of transcription; PI3K, phosphoinositide 3-kinase; PIP2, phosphatidylinositol-4,5-bisphosphate; PIP3, phosphatidylinositol 3,4,5-triphosphate; PTEN, phosphatase and tensin homolog; AKT, protein kinase B; eNOS, endothelial nitric oxide synthase; mTOR, mammalian/mechanistic target of rapamycin; AMPK, AMP-activated protein kinase; ATP/AMP, adenosine triphosphate/adenosine monophosphate; Hes1, hairy and enhancer of split 1; NICD, Notch intracellular domain; HIF-1α, hypoxia inducible factor-1α; NIX, BNIP3L.

Myocardial I/R injury was reported to be related to several pathophysiological features, including the inflammatory response, endothelial dysfunction, generation of oxygen free radicals, mitochondrial dysfunction, myocardial cell apoptosis and autophagy (24, 25). Multiple molecular mechanisms may be involved in the underlying protective mechanisms of BBR against myocardial injury, and a better understanding of these protective mechanisms will provide better insight into BBR (Figure 13).

#### Antioxidant Effect

It has been extensively reported that ischemia/reperfusion injury (I/R injury) is associated with excessive oxidative stress, and the increased consumption of antioxidants in the ischemic and reperfused myocardium results in substantial damage to the heart (26–28). One of the enzymes that contributes to oxidative stress in I/R injury is myeloperoxidase (MPO). BBR-treated rats exhibited markedly reduced myocardial MPO activity (20). BBR also exhibits direct antioxidant properties, including the induction of gp91phox protein expression and SOD activity (16, 21). Silent information regulator 1 (SIRT1) can deacetylate and activate forkhead box O (FOXO). FOXO can synthesize antioxidants, including catalase and manganese superoxide dismutase (MnSOD) to reduce oxidative damage (29, 30). SIRT1 expression and Foxo1 deacetylation are induced by BBR treatment, promoting an antioxidant effect (20).

#### Anti-inflammatory Effects

The phosphoinositide 3-kinase (PI3K)/AKT signaling pathway is involved in the protection of myocardial cells against I/R injury by regulating the inflammatory response (31). BBR acts as an anti-inflammatory agent by suppressing the PI3K/Akt signaling pathway to decrease the secretion of an array of proinflammatory cytokines/mediators (IL-6, IL-1β, and TNFα) in myocardial tissue (15, 32). The SIRT1/FOXO signaling pathway has also been shown to be involved in the protection of myocardial I/R injury by regulating the inflammatory response (20).

#### Antiapoptotic Effects

Cardiomyocyte apoptosis is one of the primary mechanisms of myocardial I/R injury. In a mouse model of myocardial I/R injury, BBR inhibited the activation of caspase-3, caspase-9, and apoptotic protease-activating factor 1 (Apaf-1) in cardiomyocytes (33). Notch1 signaling activation is reported to contribute to cardioprotection provided by ischemic preconditioning and post-conditioning by suppressing apoptosis (34). BBR treatment overactivates Notch1 and upregulates the expression of NICD and Hes1, indicating an essential role of Notch1/Hes1 signaling in the protective action of BBR (19). In addition, PI3K/Akt, AMPK and eNOS phosphorylation are also involved in the processing of BBR, suppressing myocardial apoptosis (17, 35). Emerging evidence strongly suggests that mTOR is a pivotal factor which negatively regulates autophagy and regulates cellular metabolism, survival, proliferation, and death (36). In cardiac fibroblasts, BBR treatment activates the AMPK signaling pathway and suppresses the mTOR/p70S6K signaling pathway to exert its protective effect (37, 38). In addition, BBR was reported to partly inhibit the PI3K/Akt/mTOR signaling pathway (36).

#### The Effect on Mitochondrial Dysfunction and Autophagy

Autophagy and mitophagy both are considered to be essential homeostatic pathways in cell, as genetic ablation of key autophagy and mitophagy genes is sufficient to induce the degeneration of cell (39, 40). Mitochondrial dysfunction is involved in myocardial I/R injury (41, 42). BBR pretreatment improved mitochondrial membrane potential, which was accompanied by significantly elevated mitochondrial complex I activity, suggesting that the mitochondrial dysfunction caused by I/R was significantly attenuated (17, 43). In myocardial I/R injury, induced autophagic flux might explain BBR's attenuation of mitochondrial dysfunction (44). In addition, BBR pretreatment regulates mitochondrial autophagy (mitophagy) via the HIF-1α/BNIP3 pathway, promoting mitochondrial autophagy (autophagy-related protein levels were elevated, and the number of autophagosomes increased) (22).

#### The Effect on Endoplasmic Reticulum Stress

Excessive ER stress has been associated with a number of pathological conditions, including ischemia/reperfusion injury, septic organ damage, and diabetes complications (45, 46). Previous studies have implicated JAK2/STAT3 signaling to play a key role in ameliorating myocardial I/R injury (47), and BBR was suggested to reduce myocardial ER stress by activating JAK2/STAT3 signaling (21).

### Implications

Clinically there lack routinely used drugs targeting the improvement on myocardial reperfusion so far (13); nevertheless, there is laboratory evidence on several myocardial protecting agents' potential in clinical use, including Sodium tanshinone IIA sulfonate (STS), Panax Notoginseng Saponins (PNS), Panax quinquefolium saponin (PQS), etc. Wei et al. (48) demonstrated that STS exerted cardioprotective effect on myocardial I/R injury in rat. STS serve this function by augment of antioxidant system, attenuating several consequences of myocardial ischemia including serum myocardial zymogram, cardiac function and microstructure disorder. Li et al. (49) revealed that the myocardial protective effect of PQS during I/R injury is partially mediated by inhibiting the opening of mitochondrial permeability transition pore (mPTP). Liu et al. (50) found PNS alleviated the myocardial ischemia and reperfusion injury in rats through HIF-1a/BNIP3 pathway of autophagy. In their study, the damage of cardiomyocytes and mitochondria was reduced by PNS, and it also increased the expressions of mitophagy related proteins.

Though the number of clinical trials is restricted, it is still evident that BBR has certain priority over other experimental agents according to the precious data. Qing et al. (51) reported BBR could improve the prognosis of patients with AMI and undergoing primary PCI, reducing several transcriptional factors. According to Yao, BBR protects patients from cardiac injury partly via reduction in autophagy and apoptosis. Li et al. (52) reported that BBR improved the prognosis of patients with acute cerebral ischemic stroke and diminished neurological impairment by reducing the serum level of macrophage migration inhibitory factor and interleukin-6.

Furthermore, the reasons for BBR's clinical potential extend further from it protecting effects against I/R injury. Outside China, the therapeutic role of BBR in other cardiovascular and metabolic diseases had attracted considerable attention from researchers worldwide. According to Marin-Neto's et al. (53) study, BBR could reduce systemic vascular resistance and heart rate and enhance cardiac index in patients with refractory chronic heart failure (CHF). In another study, BBR was also found to reduce the frequency and complexity of ventricular premature beats (VPBs) and increase left ventricular ejection fraction (LVEF) (54). A BBR-based nutraceuticals combination, reported Pirro et al. (55), reduced PCSK9 and plasma cholesterol levels and improved arterial stiffness in antiretroviral therapy treated HIV-infected patients. Zhang et al. (56) discovered that BBR improved the levels of fasting blood glucose (FBG), post-load blood glucose (P-LBG), HbA1c, blood lipid indicators and insulin-sensitivity, and reduced systolic blood pressure (SBP) on 106 subjects with T2DM and dyslipidemia. Meng et al. (57) evaluated that the anti-inflammatory action of BBR in ACS patients following PCI, which reduced TC and LDL-C levels as well as markers of inflammation. Additionally, BBR was found to have a tendency to induce better reduction in triglycerides and low-density lipoprotein cholesterol, but there was no statistical significance (57). In summary, BBR was found to possess multiple positive effects, including improving blood lipid profile, blood pressure, glucose level, insulin resistance, endothelial function, and systemic inflammation (57–62).

The protective effects of BBR are exerted via diverse pathways, including the inflammatory response, endothelial dysfunction, generation of oxygen free radicals, mitochondrial dysfunction, myocardial cell apoptosis and autophagy, which, from our point of view, makes it a highly promising candidate. Compared to other drugs, BBR has distinguished advantages on regulating metabolism, including blood lipids and glucose levels, which can be remarkably constructive on improving the prognosis of cardiovascular incidents after I/R injury. Although emerging evidence is pointing to the obvious advantages of BBR, however, the gap between laboratory and clinical setting cannot be overlooked.

The purpose of animal experiments is to collect information on three aspects, including understanding disease mechanisms, suggesting intervention strategies (guiding clinical trials), and examining the potential efficacy, safety, and toxicity of the interventions. However, it is difficult to translate conclusions from preclinical research to humans in clinical trials due to the low predictive value of animal experiments (63). Some deficiencies in poor methodological quality and significant design differences between experimental animal studies and clinical trials might account for the low predictive value of animal experiments with weak internal and external validity (64, 65). Systematic reviews (SRs) and meta-analyses of experimental animal studies has been suggested to facilitate the translation of research findings from animals to humans. SRs are effective in identifying these factors in the short term and promoting their avoidance in the long term (66). In our study, although we found that BBR exerted cardioprotective functions in myocardial I/R injury, the included studies had a high risk of selection, performance and detection bias, and negative findings were less likely to be published. These factors may impair the reliability of pre-clinical evidence of BBR's protective effects in myocardial I/R injury. Therefore, well-designed animal studies or/and RCTs are urgently called for the evaluation of the expected effects of BBR.

### Limitations

First, our meta-analysis includes studies performed solely in China. Although we found that many studies have convincing evidence of BBR showing diverse therapeutic effects on myocardial ischemia/reperfusion injury, these studies were primarily originated from China. Therefore, it is indeed a limitation of the present study. Second, similar to other meta-analyses of preclinical studies, studies with experimental animals are most likely to be published if they present positive results. Thus, the efficacy was overestimated, which indeed contributed as a major source of bias. Third, our study only included target animal models without other comorbidities. However, the cardiovascular comorbidities of patients with myocardial I/R injury are more complicated in a clinical setting. Fourth, data from large animal models that share more similarities in pathophysiological characteristics with humans were absent. These issues substantially limit the interpretation and extension of our results to humans.

### Conclusions

The results of this preclinical systematic review indicated that BBR significantly reduces myocardial infarct size and the incidence of ventricular arrhythmia, improves cardiac function, ameliorates myocardial apoptosis, and decreasing biomarker levels of myocardial infarction. From the analysis of the underlying mechanisms of BBR's protective effects, it seems that antioxidant, anti-inflammatory, anti-apoptosis, regulation of mitochondrial dysfunction and autophagy, and reduction of endoplasmic reticulum stress are closely associated with its effects on myocardial I/R injury. Therefore, BBR demonstrates a unique effect on myocardial I/R injury in current studies. In addition, these conclusions should be interpreted with caution due to the lack of clinical trials.

## Data Availability Statement

The original contributions presented in the study are included in the article/supplementary material, further inquiries can be directed to the corresponding author/s.

## Author Contributions

QL, KZ, and CC: study conception and design. CC, JX, DL, YL, JW, X-YC, J-JL, JL, KZ, and QL: acquisition, analysis, and interpretation of data. TC, TM, JZ, and JC: article revision. QL and DL: final approval and overall responsibility for this published work. All authors contributed to the article and approved the submitted version.

## Conflict of Interest

The authors declare that the research was conducted in the absence of any commercial or financial relationships that could be construed as a potential conflict of interest.

## References

[1] Lang IM Badr-Eslam R Greenlaw N Young R Steg PG. Management and clinical outcome of stable coronary artery disease in Austria : results from 5 years of the CLARIFY registry. Wien Klin Wochenschr. (2017) 129:879–92. 10.1007/s00508-017-1248-1 28913755 PMC5860132

[2] Mozaffarian D Benjamin EJ Go AS Arnett DK Blaha MJ Cushman M. Heart disease and stroke statistics−2015 update: a report from the American heart association. Circulation. (2015) 131:e29–322. 10.1161/CIR.0000000000000157 25520374

[3] O'Gara PT Kushner FG Ascheim DD Casey DE Jr Chung MK de Lemos JA. 2013 ACCF/AHA guideline for the management of ST-elevation myocardial infarction: a report of the American college of cardiology foundation/American heart association task force on practice guidelines. J Am Coll Cardiol. (2013) 61:e78–140. 10.1161/CIR.0b013e3182742cf6 23256914

[4] Pagliaro P Moro F Tullio F Perrelli MG Penna C. Cardioprotective pathways during reperfusion: focus on redox signaling and other modalities of cell signaling. Antioxid Redox Signal. (2011) 14:833–50. 10.1089/ars.2010.3245 20649460

[5] Baines CP. How and when do myocytes die during ischemia and reperfusion: the late phase. J Cardiovasc Pharmacol Ther. (2011) 16:239–43. 10.1177/1074248411407769 21821522

[6] Hausenloy DJ Yellon DM. Myocardial ischemia-reperfusion injury: a neglected therapeutic target. J Clin Invest. (2013) 123:92–100. 10.1172/JCI62874 23281415 PMC3533275

[7] Bonnemeier H Wiegand UK Giannitsis E Schulenburg S Hartmann F Kurowski V. Temporal repolarization inhomogeneity and reperfusion arrhythmias in patients undergoing successful primary percutaneous coronary intervention for acute ST-segment elevation myocardial infarction: impact of admission troponin T. Am Heart J. (2003) 145:484–92. 10.1067/mhj.2003.174 12660672

[8] Feng X Sureda A Jafari S Memariani Z Tewari D Annunziata G. Berberine in cardiovascular and metabolic diseases: from mechanisms to therapeutics. Theranostics. (2019) 9:1923–51. 10.7150/thno.30787 31037148 PMC6485276

[9] Tan HL Chan KG Pusparajah P Duangjai A Saokaew S Mehmood Khan T. Rhizoma coptidis: a potential cardiovascular protective agent. Front Pharmacol. (2016) 7:362. 10.3389/fphar.2016.00362 27774066 PMC5054023

[10] Battu SK Repka MA Maddineni S Chittiboyina AG Avery MA Majumdar S. Physicochemical characterization of berberine chloride: a perspective in the development of a solution dosage form for oral delivery. AAPS PharmSciTech. (2010) 11:1466–75. 10.1208/s12249-010-9520-y 20842541 PMC2974104

[11] Wang K Feng X Chai L Cao S Qiu F. The metabolism of berberine and its contribution to the pharmacological effects. Drug Metab Rev. (2017) 49:139–57. 10.1080/03602532.2017.1306544 28290706

[12] Huang ZJ Zeng Y Lan P Sun PH Chen WM. Advances in structural modifications and biological activities of berberine: an active compound in traditional Chinese medicine. Mini Rev Med Chem. (2011) 11:1122–9. 10.2174/138955711797655362 22353221

[13] Zheng Q Bao XY Zhu PC Tong Q Zheng GQ Wang Y. Ginsenoside Rb1 for myocardial ischemia/reperfusion injury: preclinical evidence and possible mechanisms. Oxid Med Cell Longev. (2017) 2017:6313625. 10.1155/2017/6313625 29430282 PMC5753014

[14] Huang Z Han Z Ye B Dai Z Shan P Lu Z. Berberine alleviates cardiac ischemia/reperfusion injury by inhibiting excessive autophagy in cardiomyocytes. Eur J Pharmacol. (2015) 762:1–10. 10.1016/j.ejphar.2015.05.028 26004523

[15] Qin-Wei Z Yong-Guang LI. Berberine attenuates myocardial ischemia reperfusion injury by suppressing the activation of PI3K/AKT signaling. Exp Ther Med. (2016) 11:978–84. 10.3892/etm.2016.3018 26998023 PMC4774358

[16] Song YF. The Protective Effects of Berberine on Myocardial Ischemia-Reperfusion Injury in Rats. Jilin: Jilin University (2010).

[17] Wang Y Liu J Ma A Chen Y. Cardioprotective effect of berberine against myocardial ischemia/reperfusion injury via attenuating mitochondrial dysfunction and apoptosis. Int J Clin Exp Med. (2015) 8:14513–9. 26550442 PMC4613127

[18] Xiong ML Wei L. Cardioprotective effect of berberine on myocardial ischemia-reperfusion injury in rats. J Hubei Universi Nationali. (2009) 1:12–14. 10.3969/j.issn.1008-8164.2009.01.00322715398

[19] Yu L Li F Zhao G Yang Y Jin Z Zhai M. Protective effect of berberine against myocardial ischemia reperfusion injury: role of Notch1/Hes1-PTEN/Akt signaling. Apoptosis. (2015) 20:796–810. 10.1007/s10495-015-1122-4 25824534

[20] Yu L Li Q Yu B Yang Y Jin Z Duan W. Berberine attenuates myocardial ischemia/reperfusion injury by reducing oxidative stress and inflammation response: role of silent information regulator 1. Oxid Med Cell Longev. (2016) 2016:1689602. 10.1155/2016/1689602 26788242 PMC4691633

[21] Zhao GL Yu LM Gao WL Duan WX Jiang B Liu XD. Berberine protects rat heart from ischemia/reperfusion injury via activating JAK2/STAT3 signaling and attenuating endoplasmic reticulum stress. Acta Pharmacol Sinica. (2016) 37:354–67. 10.1038/aps.2015.136 26806299 PMC4775848

[22] Zhu N Li J Li Y Zhang Y Du Q Hao P. Berberine protects against simulated ischemia/reperfusion injury-induced H9C2 cardiomyocytes apoptosis *in vitro* and myocardial ischemia/reperfusion-induced apoptosis *in vivo* by regulating the mitophagy-mediated HIF-1α/BNIP3 Pathway. Front Pharmacol. (2020) 11:367. 10.3389/fphar.2020.00367 32292345 PMC7120539

[23] Chang W Zhang M Li J Meng Z Xiao D Wei S. Berberine attenuates ischemia-reperfusion injury via regulation of adenosine-5′-monophosphate kinase activity in both non-ischemic and ischemic areas of the rat heart. Cardiovasc Drugs Ther. (2012) 26:467–78. 10.1007/s10557-012-6422-0 23179953

[24] Mokhtari-Zaer A Marefati N Atkin SL Butler AE Sahebkar A. The protective role of curcumin in myocardial ischemia–reperfusion injury. J Cell Physiol. (2019) 234:214–22. 10.1002/jcp.26848 29968913

[25] Yang M Linn BS Zhang Y Ren J. Mitophagy and mitochondrial integrity in cardiac ischemia-reperfusion injury. Biochim Biophys Acta Mol Basis Dis. (2019) 1865:2293–302. 10.1016/j.bbadis.2019.05.007 31100337

[26] Lassnigg A Punz A Barker R Keznickl P Manhart N Roth E. Influence of intravenous vitamin E supplementation in cardiac surgery on oxidative stress: a double-blinded, randomized, controlled study. Br J Anaesth. (2003) 90:148–54. 10.1093/bja/aeg042 12538369

[27] Sood R Narang AP Abraham R Arora U Calton R Sood N. Changes in vitamin C and vitamin E during oxidative stress in myocardial reperfusion. Indian J Physiol Pharmacol. (2007) 51:165–9. 18175661

[28] Chouchani ET Pell VR Gaude E Aksentijević D Sundier SY Robb EL. Ischaemic accumulation of succinate controls reperfusion injury through mitochondrial ROS. Nature. (2014) 515:431–5. 10.1038/nature13909 25383517 PMC4255242

[29] Brunet A Sweeney LB Sturgill JF Chua KF Greer PL Lin Y. Stress-dependent regulation of FOXO transcription factors by the SIRT1 deacetylase. Science. (2004) 303:2011–5. 10.1126/science.1094637 14976264

[30] Yang Y Duan W Li Y Jin Z Yan J Yu S. Novel role of silent information regulator 1 in myocardial ischemia. Circulation. (2013) 128:2232–40. 10.1161/CIRCULATIONAHA.113.002480 24218438

[31] Ma X Chen Z Wang L Wang G Wang Z Dong X. The pathogenesis of diabetes mellitus by oxidative stress and inflammation: its inhibition by berberine. Front Pharmacol. (2018) 9:782. 10.3389/fphar.2018.00782 30100874 PMC6072898

[32] Mo C Wang L Zhang J Numazawa S Tang H Tang X. The crosstalk between Nrf2 and AMPK signal pathways is important for the anti-inflammatory effect of berberine in LPS-stimulated macrophages and endotoxin-shocked mice. Antioxid Redox Signal. (2014) 20:574–88. 10.1089/ars.2012.5116 23875776 PMC3901384

[33] Wang L Ma H Xue Y Shi H Ma T Cui X. Berberine inhibits the ischemia-reperfusion injury induced inflammatory response and apoptosis of myocardial cells through the phosphoinositide 3-kinase/RAC-α serine/threonine-protein kinase and nuclear factor-κB signaling pathways. Exp Ther Med. (2018) 15:1225–32. 10.3892/etm.2017.5575 29403554 PMC5780743

[34] Zhou XL Wan L Xu QR Zhao Y Liu JC. Notch signaling activation contributes to cardioprotection provided by ischemic preconditioning and postconditioning. J Transl Med. (2013) 11:251. 10.1186/1479-5876-11-251 24098939 PMC3853230

[35] Chen K Li G Geng F Zhang Z Li J Yang M. Berberine reduces ischemia/reperfusion-induced myocardial apoptosis via activating AMPK and PI3K-Akt signaling in diabetic rats. Apoptosis. (2014) 19:946–57. 10.1007/s10495-014-0977-0 24664781

[36] Chen H Ji Y Yan X Su G Chen L Xiao J. Berberine attenuates apoptosis in rat retinal müller cells stimulated with high glucose via enhancing autophagy and the AMPK/mTOR signaling. Biomed Pharmacother. (2018) 108:1201–7. 10.1016/j.biopha.2018.09.140 30372821

[37] Maiti P Plemmons A Dunbar GL. Combination treatment of berberine and solid lipid curcumin particles increased cell death and inhibited PI3K/Akt/mTOR pathway of human cultured glioblastoma cells more effectively than did individual treatments. PLoS ONE. (2019) 14:e0225660. 10.1371/journal.pone.0225660 31841506 PMC6913937

[38] Ai F Chen M Yu B Yang Y Xu G Gui F. Berberine regulates proliferation, collagen synthesis and cytokine secretion of cardiac fibroblasts via AMPK-mTOR-p70S6K signaling pathway. Int J Clin Exp Pathol. (2015) 8:12509–16. 26722438 PMC4680383

[39] Hara T Nakamura K Matsui M Yamamoto A Nakahara Y Suzuki-Migishima R. Suppression of basal autophagy in neural cells causes neurodegenerative disease in mice. Nature. (2006) 441:885–9. 10.1038/nature04724 16625204

[40] Komatsu M Waguri S Chiba T Murata S Iwata J Tanida I. Loss of autophagy in the central nervous system causes neurodegeneration in mice. Nature. (2006) 441:880–4. 10.1038/nature04723 16625205

[41] Bulteau AL Lundberg KC Ikeda-Saito M Isaya G Szweda LI. Reversible redox-dependent modulation of mitochondrial aconitase and proteolytic activity during in vivo cardiac ischemia/reperfusion. Proc Natl Acad Sci USA. (2005) 102:5987–91. 10.1073/pnas.0501519102 15840721 PMC1087934

[42] Zhou H Ren J Toan S Mui D. Role of mitochondrial quality surveillance in myocardial infarction: from bench to bedside. Ageing Res Rev. (2021) 66:101250. 10.1016/j.arr.2020.101250 33388396

[43] Qin X Zhao Y Gong J Huang W Su H Yuan F. Berberine protects glomerular podocytes via inhibiting Drp1-mediated mitochondrial fission and dysfunction. Theranostics. (2019) 9:1698–713. 10.7150/thno.30640 31037132 PMC6485199

[44] M CX. The Protective Mechanisms of Berberine Pretreatment on H9C2 Cardiomyocytes During Hypoxia/Reoxygenation Injury (2014).

[45] Huang L Xie H Liu H. Endoplasmic reticulum stress, diabetes mellitus, and tissue injury. Curr Protein Pept Sci. (2014) 15:812–8. 10.2174/1389203715666140930125426 25266908

[46] Toth A Nickson P Mandl A Bannister ML Toth K Erhardt P. Endoplasmic reticulum stress as a novel therapeutic target in heart diseases. Cardiovasc Hematol Disord Drug Targets. (2007) 7:205–18. 10.2174/187152907781745260 17896961

[47] Kurdi M Booz GW. Can the protective actions of JAK-STAT in the heart be exploited therapeutically? Parsing the regulation of interleukin-6-type cytokine signaling. J Cardiovasc Pharmacol. (2007) 50:126–41. 10.1097/FJC.0b013e318068dd49 17703129

[48] Wei B Li WW Ji J Hu QH Ji H. The cardioprotective effect of sodium tanshinone IIA sulfonate and the optimizing of therapeutic time window in myocardial ischemia/reperfusion injury in rats. Atherosclerosis. (2014) 235:318–27. 10.1016/j.atherosclerosis.2014.05.924 24911635

[49] Li D Liu M Tao TQ Song DD Liu XH Shi DZ. Panax quinquefolium saponin attenuates cardiomyocyte apoptosis and opening of the mitochondrial permeability transition pore in a rat model of ischemia/reperfusion. Cell Physiol Biochem. (2014) 34:1413–26. 10.1159/000366347 25301366

[50] Liu XW Lu MK Zhong HT Wang LH Fu YP. Panax notoginseng saponins attenuate myocardial ischemia-reperfusion injury through the HIF-1α/BNIP3 pathway of autophagy. J Cardiovasc Pharmacol. (2019) 73:92–9. 10.1097/FJC.0000000000000640 30531436

[51] Qing Y Dong X Hongli L Yanhui L. Berberine promoted myocardial protection of postoperative patients through regulating myocardial autophagy. Biomed Pharmacother. (2018) 105:1050–3. 10.1016/j.biopha.2018.06.088 30021340

[52] Li Y Wang P Chai MJ Yang F Li HS Zhao J. [Effects of berberine on serum inflammatory factors and carotid atherosclerotic plaques in patients with acute cerebral ischemic stroke]. Zhongguo Zhong Yao Za Zhi. (2016) 41:4066–71. 10.4268/cjcmm20162128 28929697

[53] Marin-Neto JA Maciel BC Secches AL Gallo Júnior L. Cardiovascular effects of berberine in patients with severe congestive heart failure. Clin Cardiol. (1988) 11:253–60. 10.1002/clc.4960110411 3365876

[54] Zeng X Zeng X. Relationship between the clinical effects of berberine on severe congestive heart failure and its concentration in plasma studied by HPLC. Biomed Chromatogr. (1999) 13:442–4. 10.1002/(SICI)1099-0801(199911)13:7 <442::AID-BMC908>3.0.CO;2-A 10534753

[55] Pirro M Francisci D Bianconi V Schiaroli E Mannarino MR Barsotti F. NUtraceutical TReatment for hYpercholesterolemia in HIV-infected patients. The NU-TRY(HIV) randomized cross-over trial. Atherosclerosis. (2019) 280:51–7. 10.1016/j.atherosclerosis.2018.11.026 30471555

[56] Zhang Y Li X Zou D Liu W Yang J Zhu N. Treatment of type 2 diabetes and dyslipidemia with the natural plant alkaloid berberine. J Clin Endocrinol Metab. (2008) 93:2559–65. 10.1210/jc.2007-2404 18397984

[57] Meng S Wang LS Huang ZQ Zhou Q Sun YG Cao JT. Berberine ameliorates inflammation in patients with acute coronary syndrome following percutaneous coronary intervention. Clin Exp Pharmacol Physiol. (2012) 39:406–11. 10.1111/j.1440-1681.2012.05670.x 22220931

[58] Zhang H Wei J Xue R Wu JD Zhao W Wang ZZ. Berberine lowers blood glucose in type 2 diabetes mellitus patients through increasing insulin receptor expression. Metabolism. (2010) 59:285–92. 10.1016/j.metabol.2009.07.029 19800084

[59] Chang X Wang Z Zhang J Yan H Bian H Xia M. Lipid profiling of the therapeutic effects of berberine in patients with nonalcoholic fatty liver disease. J Transl Med. (2016) 14:266. 10.1186/s12967-016-0982-x 27629750 PMC5024486

[60] Di Pierro F Putignano P Villanova N Montesi L Moscatiello S Marchesini G. Preliminary study about the possible glycemic clinical advantage in using a fixed combination of *Berberis aristata* and *Silybum marianum* standardized extracts versus only *Berberis aristata* in patients with type 2 diabetes. Clin Pharmacol. (2013) 5:167–74. 10.2147/CPAA.S54308 24277991 PMC3838471

[61] Yan HM Xia MF Wang Y Chang XX Yao XZ Rao SX. Efficacy of berberine in patients with non-alcoholic fatty liver disease. PLoS ONE. (2015) 10:e0134172. 10.1371/journal.pone.0134172 26252777 PMC4529214

[62] Wei W Zhao H Wang A Sui M Liang K Deng H. A clinical study on the short-term effect of berberine in comparison to metformin on the metabolic characteristics of women with polycystic ovary syndrome. Eur J Endocrinol. (2012) 166:99–105. 10.1530/EJE-11-0616 22019891

[63] McGonigle P Ruggeri B. Animal models of human disease: challenges in enabling translation. Biochem Pharmacol. (2014) 87:162–71. 10.1016/j.bcp.2013.08.006 23954708

[64] van der Worp HB Howells DW Sena ES Porritt MJ Rewell S O'Collins V. Can animal models of disease reliably inform human studies? PLoS Med. (2010) 7:e1000245. 10.1371/journal.pmed.1000245 20361020 PMC2846855

[65] Sena ES Currie GL McCann SK Macleod MR Howells DW. Systematic reviews and meta-analysis of preclinical studies: why perform them and how to appraise them critically. J Cereb Blood Flow Metab. (2014) 34:737–42. 10.1038/jcbfm.2014.28 24549183 PMC4013765

[66] Perel P Roberts I Sena E Wheble P Briscoe C Sandercock P. Comparison of treatment effects between animal experiments and clinical trials: systematic review. BMJ. (2007) 334:197. 10.1136/bmj.39048.407928.BE 17175568 PMC1781970

